# High Prevalence of USA300 Among Clinical Isolates of Methicillin-Resistant *Staphylococcus aureus* on St. Kitts and Nevis, West Indies

**DOI:** 10.3389/fmicb.2019.01123

**Published:** 2019-06-05

**Authors:** Luca Guardabassi, Arshnee Moodley, Andrea Williams, Marc Stegger, Peter Damborg, Iona Halliday-Simmonds, Patrick Butaye

**Affiliations:** ^1^ Department of Veterinary and Animal Sciences, Faculty of Health and Medical Sciences, University of Copenhagen, Copenhagen, Denmark; ^2^ Department of Biomedical Sciences, Ross University School of Veterinary Medicine, Basseterre, Saint Kitts and Nevis; ^3^ Department of Pathobiology and Population Sciences, The Royal Veterinary College, Hatfield, United Kingdom; ^4^ Department of Microbiology, Joseph N France General Hospital, Basseterre, Saint Kitts and Nevis; ^5^ Department of Bacteria, Parasites, and Fungi, Statens Serum Institut, Copenhagen, Denmark; ^6^ Department of Pathology, Bacteriology and Poultry Diseases, Faculty of Veterinary Medicine, Ghent University, Merelbeke, Belgium

**Keywords:** clinical, MRSA, antimicrobial resistance, whole genome sequencing, USA300

## Abstract

Limited information is available on antimicrobial susceptibility and clonal distribution of *Staphylococcus aureus* in the Caribbean region. The aims of this study were to determine the prevalence of antimicrobial resistance among *S. aureus* isolates and to reveal the frequency and population structure of methicillin-resistant *S. aureus* (MRSA) in St. Kitts and Nevis, a small island country in the West Indies. A total of 152 *S. aureus* isolates were collected from consecutive samples submitted to the clinical microbiology laboratory of the main referral hospital from March 2017 to January 2018. Samples came from all units in the hospital and a small number came from external submissions, and comprised a total of 119 clinical specimens and 33 nasal swabs collected from staff and patients. All *S. aureus* isolates were confirmed by matrix-assisted laser desorption/ionization time-of-flight mass spectrometry. Minimal Inhibitory Concentrations (MICs) of clinically relevant antimicrobials were determined by broth microdilution, and diversity of MRSA isolates was assessed by whole genome sequencing (WGS) analysis. MRSA accounted for 45% (69/152) of the isolates. The highest rates of resistance to non-β-lactam agents were observed for erythromycin (55%), moxifloxacin (41%), and levofloxacin (40%), whereas resistance to the other drugs tested was ≤6%. All isolates were susceptible to ceftaroline, linezolid, teicoplanin, telavancin, and vancomycin. WGS-based multilocus sequence typing (MLST) showed that approximately 88% of the MRSA isolates belonged to ST8. Phylogenetic analysis on 801 single-nucleotide polymorphisms (SNPs) identified among the MRSA ST8 isolates indicates a large degree of genetic diversity. However, all ST8 strains clustered within the distinct clade that defines the USA300 North American Epidemic lineage (Panton-Valentine Leukocidin (PVL) +, arginine catabolic mobile element (ACME) +, Staphylococcal cassettes chromosome *mec* IVa (SCC*mec* IVa)). Our data show high levels of methicillin, macrolide, and fluoroquinolone resistance among *S. aureus* on St. Kitts and Nevis. The USA300 North American epidemic lineage is responsible for the vast majority of MRSA infections on this Caribbean island.

## Introduction

*Staphylococcus aureus* is an opportunistic pathogen that causes a variety of infections ranging from mild skin and soft tissue infections to severe bacteremia and pneumonia. Antimicrobial resistance is a great concern in this species because of the global spread of methicillin-resistant *Staphylococcus aureus* (MRSA) that are resistant to all conventional β-lactams and may display additional resistance to non-β-lactams. A few pandemic MRSA clones are distributed worldwide, whereas others predominate in restricted geographical areas (Monecke et al., 2011). Molecular epidemiological studies aimed at understanding local patterns of antimicrobial resistance, MRSA prevalence, and clonal distribution are essential to implement measures for control and treatment of *S. aureus* infections.

There is limited information on antimicrobial susceptibility and genetic diversity of *S. aureus* in the Caribbean, a major destination of international tourism. The current knowledge is limited to studies from Trinidad and Tobago (Akpaka et al., 2006, 2007, 2011; Orrett and Land, 2006; Monecke et al., 2014) and sporadic reports from Cuba (Hopman et al., 2012), Dominican Republic and Martinique (Uhlemann et al., 2012), Jamaica (Brown, 2015), and Puerto Rico (Rodríguez et al., 2002; Morales-Torres et al., 2015). It has been shown that the majority of strains from this region belong to Clonal Complex (CC) 8 (Akpaka et al., 2017; Strauß et al., 2017).

The aims of this study were to determine the prevalence of antimicrobial resistance among *S. aureus* isolates and to reveal the frequency and population structure of MRSA in St. Kitts and Nevis, a small island country in the West Indies.

## Materials and Methods

### Ethics

The Interim Ethics Review Committee (IERC) of the Federation of Saint Christopher and Nevis has reviewed the protocol and based on the findings approved the research project. The IERC approval code is 2017-11-007.

### Bacterial Isolates

*S. aureus* isolates were obtained from consecutive samples submitted between March 2017 and January 2018, to the clinical microbiology laboratory at Joseph N France General Hospital, St. Kitts. The samples from which *S. aureus* was isolated included 119 clinical specimens and 33 nasal swabs from hospital staff (*n* = 29) and patients (*n* = 4). *S. aureus* was presumptively identified by colony morphology, catalase and coagulase test at the hospital laboratory, and later confirmed at the University of Copenhagen by matrix-assisted laser desorption/ionization time-of-flight mass spectrometry (MALDI-TOF MS, Vitek MS RUO, bioMérieux, France) using Saramis v.3.5 (bioMérieux) for spectral interpretation. Antimicrobial susceptibility was tested by broth microdilution using the EUSTAPF commercial panel (Thermo Fisher Scientific, Denmark) according to the methodology and breakpoints specified by the European Committee on Antimicrobial Susceptibility testing (EUCAST^1^). Clinical breakpoints were available for all drugs, except mupirocin for which we used the epidemiological cutoff. Isolates falling in the intermediate category were considered as resistant when reporting data on prevalence of resistance. Methicillin resistance was assessed using a multiplex PCR as described by Stegger et al. (2012).

### Whole Genome Sequencing and Analysis of Methicillin-Resistant *S. aureus* Isolates

Overnight cultures were grown in tryptic soy broth at 37°C with 200-rpm shaking. Genomic DNA from the MRSA strains was extracted using the DNeasy Blood and Tissue kit (Qiagen). Library preparation was carried out using the Nextera XT kit and paired end 2 × 250 bp sequencing on a MiSeq, all following standard Illumina protocols (Illumina, Inc., United States). All raw reads have been deposited in the European Nucleotide Archive under study accession number PRJEB29509.

Initial analyses (*de novo* assembly and multilocus sequence type (MLST) determination) were performed in CLC Genomics Workbench v.11.0 using the tools within the Microbial Genomics Module. The ResFinder v.3.0, VirulenceFinder v.1.5, and SCC*mec*Finder v1.2 Web-based pipelines at the Centre for Genomic Epidemiology^2^ were used to search for the presence of known virulence and antibiotic resistance genes, and to determine the Staphylococcal Cassette Chromosome *mec* (SCC*mec*) types (Zankari et al., 2012; Joensen et al., 2014; Kaya et al., 2018). To investigate the genetic relatedness of the St. Kitts and Nevis ST8 isolates, a rooted maximum likelihood tree was generated from core genome SNPs, performed as previously described by Strauß et al. (2017). Briefly, sequence reads were mapped against the reference chromosome of *S. aureus* TCH1516, a USA300 ST8 strain (GenBank accession no. CP000730), using NASP v.1.0.0 (Highlander et al., 2007). First, duplicated regions in the reference were removed using NUCmer followed by aligning of the reads using the Burrows–Wheeler Aligner (Li and Durbin, 2009). All positions with <10-fold coverage or if the variant was present in <90% of the base calls were excluded. Indications of putative horizontal gene transfer events were purged from the alignment using Gubbins v.2.3 (Croucher et al., 2014). The relatedness of the isolates was inferred using PHyML v.3.0 (Guindon et al., 2010) with Smart Model Selection (Lefort et al., 2017) under the Bayesian Information Criterion with 100 bootstrap replicates. Additionally, a second rooted phylogeny of the St. Kitts ST8 strains against a global ST8 strain collection (Strauß et al., 2017) was constructed similarly as described above.

## Results

A total of 152 *S. aureus* were isolated at the clinical microbiology laboratory of Joseph N France General Hospital between March 2017 and January 2018. The most common source of isolation was pus (*n* = 53), followed by nasal cavity (*n* = 33), wounds (*n* = 25), catheters (*n* = 11), blood (*n* = 7), skin (*n* = 4), urine (*n* = 3), and others (*n* = 16). Thirty-three nasal isolates were obtained from screening hospital (*n* = 29) personnel and patients (*n* = 4), and only one strain per individual was included. The isolates originated from the surgical ward (*n* = 30), accident and emergency (*n* = 8), hemodialysis (*n* = 17), nursery (*n* = 4), pediatric (*n* = 14), intensive care unit (*n* = 3), operation theatre (*n* = 1), medical ward (*n* = 9), and orthopedics (*n* = 36). There were 25 isolates from patients in the doctors’ office consultation rooms and 5 isolates from patients in private practice rooms at the hospital (Tables 1 and 2).

**Table 1:** Origin of the samples from which total *S. aureus* and MRSA were isolated.

Origin	Total *S. aureus*	MRSA
Surgical ward	30	19
Orthopedics	36	10
Accident and emergency	8	3
Doctors office consultation rooms	25	14
Hemodialysis	17	2
Nursery	4	3
Pediatric	14	8
Intensive care unit	3	2
Private practices in clinic	5	5
Operation theatre	1	1
Medical	9	3
Total	152	69

**Table 2:** Numbers of *S. aureus* and MRSA isolated from each sample type.

Sample type	*S. aureus*	MRSA
Blood	7	3
Catheter	11	0
Nasal	33	9
Pus	53	35
Wound	25	12
Skin	4	3
Urine	3	2
Other/unknown	16	5
Total	152	69

Seventy (46%) isolates were resistant to cefoxitin and 69 of them (45%) were confirmed to be MRSA by *mecA* detection. MRSA was isolated from pus (*n* = 35), nasal cavity (*n* = 9), wounds (*n* = 12), catheters (*n* = 1), blood (*n* = 3), skin (*n* = 3), urine (*n* = 2), or other/unknown samples (*n* = 4) (Table 2). MRSA-positive samples originated from the following wards: surgical ward (*n* = 19), accident and emergency (*n* = 3), hemodialysis (*n* = 1), nursery (*n* = 3), pediatric, (*n* = 8), intensive care unit (*n* = 3), operation theatre (*n* = 1), medical (*n* = 3), and orthopedics (*n* = 1). There were 14 MRSA isolates from doctors’ office consultation rooms and 5 MRSA isolates came from private practice rooms. The consultation rooms (doctors’ and private) can be considered as outpatient samples. However, we cannot exclude outpatients in other wards (e.g., the hemodialysis unit) (Table 1). The prevalence of resistance to non-β-lactam agents was high for daptomycin (130/152, 86%) erythromycin (81/152, 53%), moxifloxacin (58/152, 38%), and levofloxacin (58/152, 38%). Lower proportions of resistant isolates were seen for tetracycline (7/152, 5%), tobramycin (7/152, 5%), clindamycin (5/152, 3%), fusidic acid (5/152, 3%), gentamicin (3, /152, 2%), mupirocin (2/152, 1%), and trimethoprim/sulfamethoxazole (2/152, 1%). A single isolate was resistant to rifampicin. All isolates were susceptible to ceftaroline, linezolid, teicoplanin, telavancin, and vancomycin (Table 3).

**Table 3:** Percentages of antimicrobial resistance in methicillin-susceptible (MSSA) and methicillin-resistant *S. aureus* (MRSA).

Antimicrobial	Resistance %		
	All *S. aureus* (*n* = 152)	MSSA (*n* = 82)	MRSA (*n* = 69)
Cefoxitin	46.0	1.2	100
Ceftaroline	0	0	0
Clindamycin	3.3	3.6	2.9
Daptomycin	85.5	75.9	97.1
Erythromycin	53.3	21.7	91.3
Fusidic acid	3.3	2.4	4.3
Gentamicin	2	0	4.3
Levofloxacin	38.2	7.2	75.4
Linezolid	0	0	0
Moxifloxacin	38.2	8.4	73.9
Rifampicin	0.7	0	1.4
Teicoplanin	0	0	0
Telavancin	0	0	0
Tetracycline	4.6	0	10.1
Tobramycin	4.6	0	10.1
Trimethoprim/sulfamethoxazole	1.3	2.4	0
Vancomycin	0	0	0
Mupirocin	1.3	0	2.9

**Figure 1:**
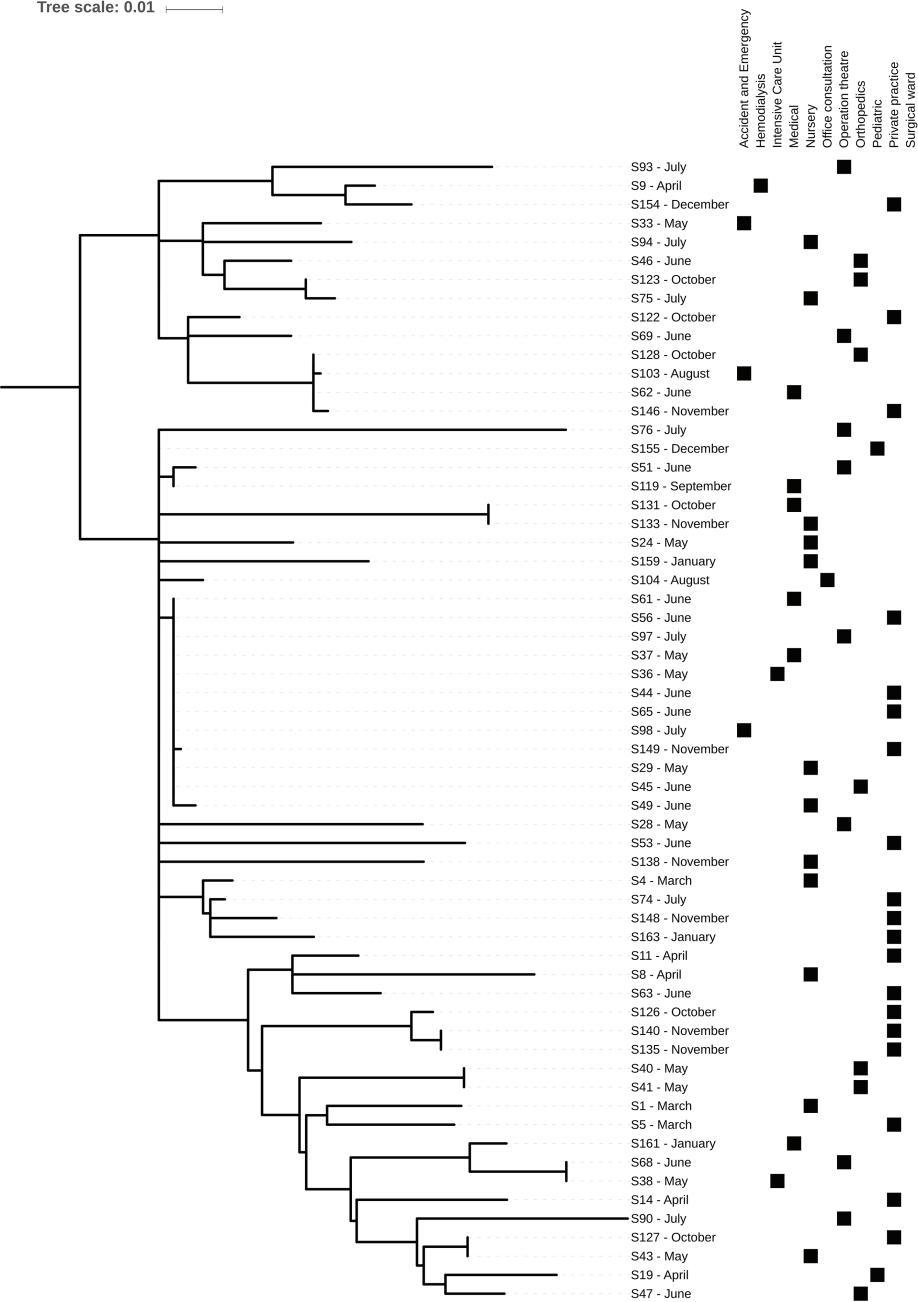
Midpoint-rooted core genome phylogeny of the 61 St. Kitts and Nevis ST8 MRSA isolates using the chromosome of methicillin-resistant *S. aureus* TCH1516, a USA300 reference strain. Phylogeny was based on an alignment of 801 SNPs. Metadata describing sample month and origins is provided.

*In silico* MLST based on WGS analysis of the 69 confirmed MRSA isolates showed a high prevalence of ST8 (61/69, 88%). The remaining isolates belonged to ST5 (*n* = 2), ST4080 (*n* = 2), ST30 (*n* = 1), ST72 (*n* = 1), ST121 (*n* = 1), and ST134 (*n* = 1). SNP-based analysis on the 61 ST8 isolates showed that these isolates differed in 801 core genome SNPs, indicating a high genetic diversity, with the majority of strains resolving into three clusters (Figure 1). In seven instances, identical or closely related (2–6 SNP differences) strains were observed. In two cases, two different patients in the same ward and month were infected with the same strain (S40/S41 and S135/S140). In three cases, two patients from different wards (S43/S127; S38/S68; S131/S133) and in one other case four (S62/S103/S128/S146) patients from different wards and eventually different times harbored either identical or closely related isolates (up to four SNP differences). The remaining cluster consisted of 12 skin or mucosa-associated strains, isolated from 7 wards and mainly between May and July from both inpatients and outpatients (Figure 1). Most of the MRSA isolates from nasal swabs (*n* = 9) originated from hospital personnel (*n* = 8) and included six ST8, one ST212, and one ST834 strains. The remaining nasal strain was isolated from a patient in ICU and belonged to ST8. The ST8 isolates were scattered across the phylogeny without any apparent epidemiological relationship except one strain from a nurse, which was also found in a blood culture from an ICU patient indicating a possible link. However, the patient strain was isolated 1 month before the nurse strain.

**Figure 2:**
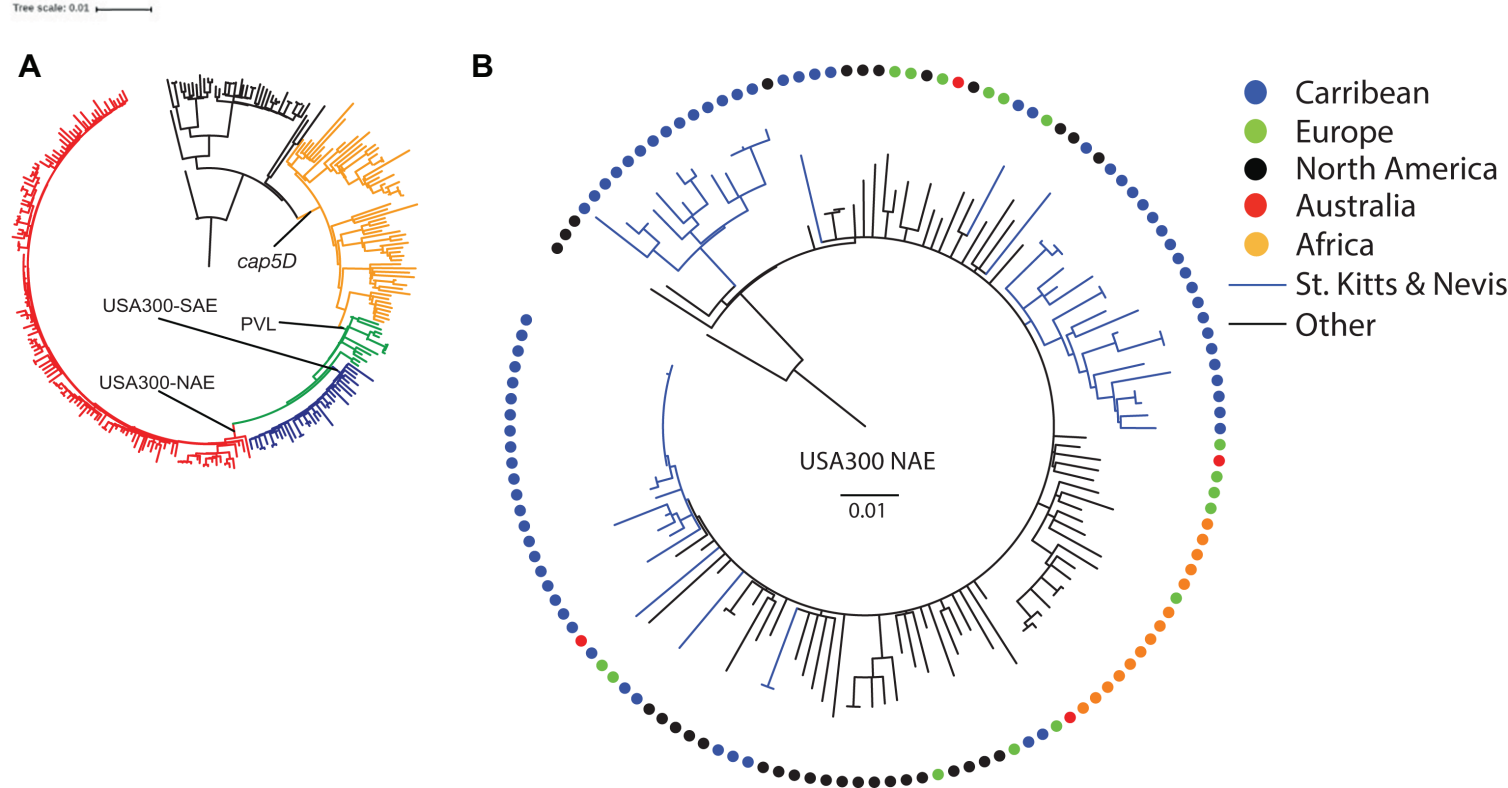
Rooted phylogenies of **(A)** *S. aureus* ST8 isolates from St. Kitts and Nevis and from a global, representative collection (*n* = 224) described by Strauß et al. (2017), and **(B)** a detailed presentation of the 135 isolates belonging to the USA300-NAE lineage. Colored circles depict geographic region of origin. The scale bar indicates the substitution rate.

Nearly all isolates carried genes encoding resistance to streptomycin [*ant(6)-Ia]*, amikacin and other aminoglycosides [*aph(3')-III]*, fosfomycin (*fosD*), macrolides, lincosamides and streptogramins [*mph*(C) and *mrs*(A)], and penicillin (*blaZ*). Genes encoding resistance to trimethoprim (*dfrG*) and phenicols (*cat*) were found in four and one isolate, respectively. A second set core genome SNP phylogenies based on 8,859 variable positions was performed containing the St. Kitts and Nevis ST8 isolates and a global, representative collection of 224 ST8s (Strauß et al., 2017) to determine the origins of our strains (Figure 2A). All our ST8 isolates clustered exclusively within the USA300-NAE lineage (1,995 SNPs, Figure 2B), with the majority of the isolates (85%) found within three St. Kitts and Nevis-specific clusters.

## Discussion

This is the first study providing insights into antimicrobial resistance and clonal distribution of *S. aureus* in St. Kitts and Nevis, and one of the few studies using WGS to understand the population structure within the Caribbean region. A high proportion of clinical isolates from this Caribbean island were resistant to methicillin, macrolides, and fluoroquinolones, mainly due to the widespread occurrence of USA300-NAE. This epidemic clone has been recently reported as the most frequent in other Caribbean countries, namely Cuba (Hopman et al., 2012), Martinique (Uhlemann et al., 2012), and Trinidad and Tobago (Monecke et al., 2014). Notably, we did not detect ST239-MRSA-III that was virtually the only MRSA clone circulating in Trinidad and Tobago until 2004 (Akpaka et al., 2007) and is still the most prevalent MRSA type in this country according to a recent study (Monecke et al., 2014). Similarly, we isolated a single strain belonging to ST30-MRSA-V, another PVL-positive clone that was recently reported as predominant in the Dominican Republic and rare in Martinique (Uhlemann et al., 2012). The latter clone was undetected in Trinidad and Tobago (Monecke et al., 2014). Altogether, these data indicate the occurrence of geographical differences in the prevalence of MRSA clones within the Caribbean region.

The frequency of MRSA among *S. aureus* isolated at the main referral hospital in St. Kitts and Nevis was much higher than the MRSA isolation frequencies reported in other Caribbean countries (12–39%) (Akpaka et al., 2006; Orrett and Land, 2006; Uhlemann et al., 2012; Monecke et al., 2014). However, these studies are not easily comparable since they were conducted in different years and used different methods for sample selection and strain isolation. Similar to isolates from Trinidad and Tobago (Akpaka et al., 2006; Orrett and Land, 2006) and Martinique (Uhlemann et al., 2012), MRSA isolates from St. Kitts and Nevis displayed high (≥40%) percentages of resistance to macrolides and fluoroquinolones. However, they were usually susceptible to older drugs such as tetracyclines, gentamicin, and potentiated sulfonamides as well as topical antimicrobials such as fusidic acid and mupirocin, which can be usefully employed for managing skin and wound infections. We observed one cefoxitin-resistant strain (MIC >8 μg/mL) that was *mecA-* and *mecC-*negative upon PCR testing. This phenomenon has previously been described and β-lactam resistance was attributed to mutations in various genes including those encoding penicillin-binding proteins (Banerjee et al., 2010; Ba et al., 2014). This strain needs further investigation to confirm the mechanism of resistance. Surprisingly we had a very high prevalence of resistance against daptomycin. Most strains had an MIC of 2 μg/ml, which is just above the EUCAST breakpoint (Humphries et al., 2013). There is no clear explanation for this high level of resistance and this needs further investigation.

None of the MRSA isolates from St. Kitts belonged to livestock-associated MRSA lineages. Recently, the occurrence of MRSA was reported in pigs in Trinidad (Gordon et al., 2014) and the livestock-associated clone ST398 has been associated with human infections in the Dominican Republic and Martinique (Uhlemann et al., 2012). The apparent lack of this and other livestock-associated MRSA clones among human clinical isolates in St. Kitts and Nevis may reflect the limited size of livestock production in this country, which largely depends on import of meat and other food animal products.

The genome analyses revealed that all the ST8 isolates belonged to the USA300-NAE lineage. Within this lineage, the majority (85%) of the isolates fell into three specific clusters comprising only isolates from St. Kitts and Nevis. Overlaying information of geographical origin of all USA300-NAE isolates did not indicate any common source of the USA300-NAE, mainly because of the radial topology observed within this clade. This Caribbean island is a tourist hub receiving many cruise ships with passengers visiting the island for a day but with longer stay-tourism increasing. Moreover, there is a large influx of students and residents from the US, and the local population has many contacts with the US as many have family members living there. This close contact may explain the diversity seen within ST8 as new strains may have been introduced on multiple occasions, and then evolved into three distinct island-specific USA300-NAE sub-lineages.

We observed identical strains in patients from the same ward during the same month as well as in patients in different wards at different times. One cluster of 12 strains that were very similar was detected over a 3-month period during the hot season, and spanned seven different wards. It is unclear if this was an outbreak.

The major limitation of this study is that it is based on passive surveillance. The actual community prevalence of MRSA in the population of St. Kitts and Nevis is unknown. Furthermore, our study population could be subjected to sampling bias since patients were not routinely sampled for microbiological analysis. All samples were obtained from the island’s main hospital, which acts as a referral laboratory for the other hospitals in the country. This could lead to an overestimation of MRSA among *S. aureus* isolates.

In conclusion, almost half of the *S. aureus* infections recorded at the Joseph N France General Hospital in St. Kitts and Nevis are caused by MRSA. Most of the MRSA strains (88%) belong to the multidrug-resistant USA300-NAE lineage, with the presence of three major country-specific sub-lineages circulating in this Caribbean island country.

## Ethics Statement

This study was approved by the IERC of St. Kitts and Nevis, with code IERC-2017-11-007.

## Author Contributions

The study was conceived by LG and PB. AW was responsible for the collection of the strains and initial susceptibility testing. IH-S was responsible for further biochemical analysis of the strains and confirmation testing. AM, MS and PD were responsible for the WGS and analysis of the data. All authors contributed to the writing of the manuscript.

### Conflict of Interest Statement

The authors declare that the research was conducted in the absence of any commercial or financial relationships that could be construed as a potential conflict of interest.

The handling editor declared a past co-authorship with the authors MS and LG.

## Footnotes

^1^www.eucast.org

^2^http://www.genomicepidemiology.org/
